# Progranulin haploinsufficiency disrupts cathepsin D function, resulting in lysosomal abnormalities and elevated TDP-43 levels in the mouse brain

**DOI:** 10.3389/fnmol.2026.1970154

**Published:** 2026-10-27

**Authors:** Raheem Fazal, Arun Kumar Tharkeshwar, Sander Beel, Annet Storm, Begga Schevenels, Sandra O. Tomé, Dietmar R. Thal, Ludo Van Den Bosch, Jimmy Beckers, Philip Van Damme

**Affiliations:** 1 Laboratory of Neurobiology, Department of Neurosciences, Experimental Neurology and Leuven Brain Institute (LBI), KU Leuven—University of Leuven, Leuven, Belgium; 2 Department of Human Genetics, University of Leuven, KU Leuven, Leuven, Belgium; 3 KU Leuven Institute for Single Cell Omics (LISCO), University of Leuven, KU Leuven, Leuven, Belgium; 4 Laboratory for Neuropathology, Department of Imaging and Pathology, Leuven Brain Institute (LBI), KU Leuven—University of Leuven, Leuven, Belgium; 5 Department of Pathology, University Hospitals Leuven, Leuven, Belgium; 6 Laboratory of Neurobiology, VIB-KU Leuven Center for Neuroscience (CNL), Leuven, Belgium; 7 Department of Neurology, University Hospitals Leuven, Leuven, Belgium

**Keywords:** cathepsin D, frontotemporal dementia, lysosomes, neurodegeneration, progranulin, TDP-43

## Abstract

**Introduction:**

Frontotemporal dementia (FTD) is a leading cause of early-onset neurodegeneration, frequently linked to mutations in the *GRN* gene, which encodes the protein progranulin (PGRN). PGRN is known to play a crucial role in lysosomal function, and its deficiency contributes to FTD pathogenesis. Here, we examined the consequences of PGRN loss on lysosomal homeostasis, focusing on the lysosomal protease cathepsin D (CTSD) and TAR DNA-binding protein 43 (TDP-43) aggregation, hallmarks of FTD and related neurodegenerative diseases.

**Methods:**

Using mouse models with homozygous and heterozygous *GRN* deletions and double knockouts for *GRN* and *CTSD*, we assessed lysosomal function, CTSD expression and activity, lysosomal-associated membrane protein (LAMP) levels and localization, TDP-43 aggregation, and survival.

**Results:**

PGRN deficiency resulted in significant alterations in lysosomal function. Despite significantly elevated levels of mature and immature CTSD in PGRN-deficient mice, enzymatic activity was significantly reduced. PGRN deficiency also caused accumulation of lysosomal-associated membrane protein (LAMP) and perinuclear clustering of LAMP1-positive lysosomes, suggestive of disrupted lysosomal trafficking. Correspondingly, insoluble TDP-43 levels significantly increased, implicating lysosomal impairment in defective protein degradation and aggregation. Remarkably, double-knockout mice lacking both PGRN and CTSD exhibited significantly shortened lifespans, highlighting a potential functional interaction between these proteins in maintaining neuronal health and longevity.

**Discussion:**

These findings underscore the importance of PGRN as a central regulator of lysosomal function and protein homeostasis. Together, the data provide mechanistic insight into PGRN-dependent lysosomal biology and identify pathways that may contribute to *GRN*-associated FTD and other neurodegenerative disorders.

## Graphical abstract

**Figure:**
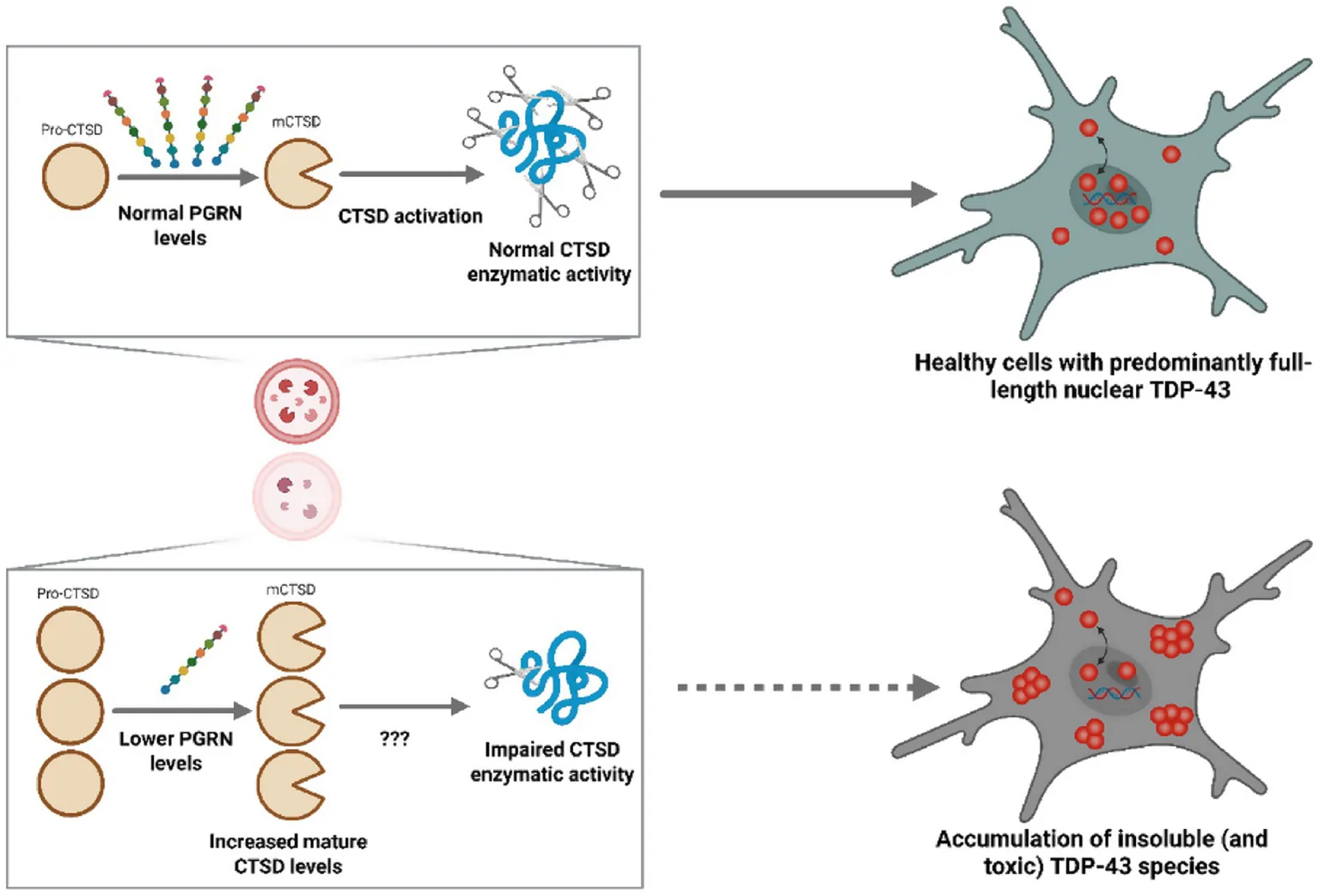
An overview of the results obtained from this study.

## Introduction

Frontotemporal dementia (FTD) stands as the second most prevalent form of early-onset dementia among individuals under 65 years and characterized by progressive degeneration of the frontal and anterior temporal lobes, leading to behavioral and/or language deficits (Grossman et al., 2023; Meeter et al., 2017; Neary et al., 2005; Olney et al., 2017; Wagner et al., 2021). Up to 40% of FTD cases are familial, reflecting a strong genetic contribution (Bang et al., 2015). Mutations in *MAPT* (microtubule-associated protein tau), *GRN* (granulin), and *C9orf72* (Chromosome 9 Open Reading Frame 72) account for ~80% of familial cases, while other disease-associated mutations occur less frequently (Bang et al., 2015; Menden et al., 2023; Moore et al., 2020; Snowden, 2023; Wagner et al., 2021). *MAPT* mutations lead to tau accumulation, whereas *GRN* and *C9orf72* mutations are associated with TAR DNA-binding protein 43 (TDP-43) aggregation. In addition, *C9orf72* mutations give rise to dipeptide repeat proteins (DPRs) and RNA foci (Antonioni et al., 2023). Despite these insights, the precise mechanisms linking gene mutations to neurodegeneration remain poorly understood, hindering the development of effective therapies.

Haploinsufficiency caused by heterozygous loss-of-function mutations in the *GRN* gene invariably results in FTD with ubiquitinated TDP-43-positive inclusions (Baker et al., 2006; Cruts et al., 2006). This reduces circulating progranulin (PGRN) levels by ~50% in plasma and cerebrospinal fluid (Ghidoni et al., 2008; Sleegers et al., 2009; Van Damme et al., 2008). However, the mechanism by which reduced PGRN levels drive neurodegeneration remains unclear. PGRN is encoded by the *GRN* gene and is an evolutionarily conserved, multifunctional, cysteine-rich glycoprotein involved in diverse biological processes [reviewed in detail by Eriksen and Mackenzie (2008) and Terryn et al. (2021)]. These include cell division, inflammation, tumor growth, wound healing, lysosomal homeostasis neurodevelopment, and neurodegeneration (Eriksen and Mackenzie, 2008; Terryn et al., 2021). Synthesized as a 68.5 kDa protein, PGRN undergoes substantial glycosylation and is secreted as a 90 kDa protein comprising 7.5 repeats of cysteine-rich granulin motifs. PGRN interacts with numerous receptors, including Tumor Necrosis Factor-alpha (TNF-α) receptor (Tang et al., 2011), sortilin (SORT1) (Hu et al., 2010), sortilin-related VPS10 domain containing receptor 2 (SORCS2) (Thomasen et al., 2023), and ephrin type-A receptor 2 (EphA2) (Neill et al., 2016). These interactions mediate diverse functions such as regulation of extracellular PGRN levels, axonal outgrowth, modulation of inflammatory responses, and capillary morphogenesis (Neill et al., 2016; Tang et al., 2011). In most cases, PGRN binds to its target proteins through epidermal growth factor (EGF)-like or cysteine-rich domains (Cui et al., 2019). PGRN is trafficked to the lysosome via prosaposin (PSAP) and receptors, including mannose 6-phosphate receptor (M6PR), and the low-density lipoprotein receptor-related protein 1 (LRP1) (Simon et al., 2023; Zhou et al., 2015). Inside the lysosomal lumen, PGRN gets cleaved into individual granulin peptides by several serine proteases (Simon et al., 2023; Zhou et al., 2015). Whether lysosomal functions are mediated by full-length PGRN, individual granulin peptides, or both remains an open question.

Growing evidence indicates that PGRN is critical for lysosomal homeostasis rather than solely being degraded (Kao et al., 2017; Paushter et al., 2018; Simon et al., 2023). Notably, PGRN deficiency has been associated with altered function of cathepsin D (CTSD), a lysosomal aspartic protease that degrades long-lived, misfolded proteins and regulates the activity of enzymes and growth factors (Beel et al., 2017; Butler et al., 2019; Valdez et al., 2017; Zhou et al., 2017b; Benes et al., 2008; Mijanovic et al., 2021). Dysfunctional lysosomal proteolysis is further evidenced by TDP-43 aggregation, observed in *post-mortem* samples of nearly 50% of all FTD subtypes (Arai et al., 2006; Neumann et al., 2006). Among individuals with FTD caused by GRN mutations, however, TDP-43 pathology is consistently observed, indicating a strong association between PGRN deficiency and TDP-43 proteinopathy (Arai et al., 2006; Neumann et al., 2006). TDP-43 is a nuclear DNA/RNA-binding protein essential for RNA processing, and its mislocalization or aggregation is deleterious to neuronal function (Ling et al., 2013).

In the present study, we used mouse models to investigate the relationship between PGRN deficiency and lysosomal dysfunction. Our results demonstrate that loss of PGRN alters CTSD expression and activity, increases LAMP1/2 levels, and disrupts lysosomal function. Furthermore, both homozygous and heterozygous *GRN* deletions result in elevated levels of insoluble TDP-43, supporting a critical role for PGRN in maintaining lysosomal homeostasis and protein quality control in the brain.

## Results

### PGRN deficiency results in reduced CTSD activity despite increased CTSD expression in mouse brain tissue

To investigate the impact of PGRN deficiency on CTSD expression and activity, we examined brain tissue from multiple transgenic mice. The genotypes of these mice include wild-type (WT) (PGRNntg – CTSDntg), heterozygous CTSD single knockout (PGRNntg – CTSD+/−), homozygous PGRN single knockout (PGRN−/− – CTSDntg), double knockout of homozygous PGRN and heterozygous CTSD (PGRN−/− – CTSD+/−), heterozygous PGRN single knockout (PGRN+/− – CTSDntg), and heterozygotes of heterozygous PGRN and heterozygous CTSD (PGRN+/− – CTSD+/−). Given that PGRN-associated neurodegenerative conditions share features with lysosomal storage disorders such as neuronal ceroid lipofuscinosis (NCL) (Kamate et al., 2019; Valdez et al., 2017), we assessed whether PGRN deficiency alters CTSD expression and enzymatic activity using western blot analysis.

Two-way ANOVA revealed significant main effects of both the PGRN genotype, *F*(2, 30) = 24.18, *p* < 0.0001 and the CTSD genotype, *F*(1, 30) = 5.648, *p* = 0.0241 on mature CTSD (mCTSD) levels. There was no significant PGRN × CTSD interaction, *F*(2, 30) = 0.07194, *p* = 0.9307. *Post hoc* multiple group analyses demonstrated a significant increase in mature CTSD levels in homozygous PGRN knockout mice (PGRN−/− – CTSDntg and PGRN−/− – CTSD+/−), compared to WT controls (*p* = 0.0002 and *p* = 0.0053 respectively) (Figures 1A,B). Similarly, analysis of immature CTSD (proCTSD) levels revealed significant main effects of both the PGRN genotype, *F*(2, 30) = 71.16, *p* < 0.0001 and the CTSD genotype, *F*(1, 30) = 25.68, *p* < 0.0001 on immature CTSD levels. However, no significant interaction was found between PGRN and CTSD genotypes *F*(2, 30) = 2.563, *p* = 0.0938. *Post hoc* multiple group analyses demonstrated a significant increase in immature CTSD levels in both homozygous PGRN knockout mice (PGRN−/− – CTSDntg and PGRN−/− – CTSD+/−), compared to WT controls (*p* < 0.0001 and *p* = 0.0005 respectively) (Figures 1A,C), indicating a consistent upregulation of CTSD expression upon PGRN loss. Of note, despite only observing a nonsignificant trend towards decreased (immature) CTSD expression in heterozygous CTSD single knockout (PGRNntg – CTSD+/−) mice when compared to WT mice (*p* = 0.2491), we did observe a significant downregulation of proCTSD upon heterozygous CTSD single knockout in both heterozygous and homozygous PGRN knockout backgrounds. Controls (*p* = 0.0365 and *p* = 0.0017 respectively) (Figures 1A,C). Interestingly, upon analysis of mature to immature CTSD ratios (Figure 1D), no significant main effects of either PGRN (*F*(2, 30) = 1.854, *p* = 0.1741) nor CTSD (*F*(1, 30) = 0.00331, *p* = 0.9545) or interaction of both genotypes (*F*(2, 30) = 1.311, *p* = 0.2845) was detected. Similarly, *Post hoc* analysis revealed that the ratio of mature to immature CTSD remained unchanged relative to WT (Figure 1D), suggesting that major dysregulations in the processing of CTSD are not evident under the conditions we examined.

**Figure 1:**
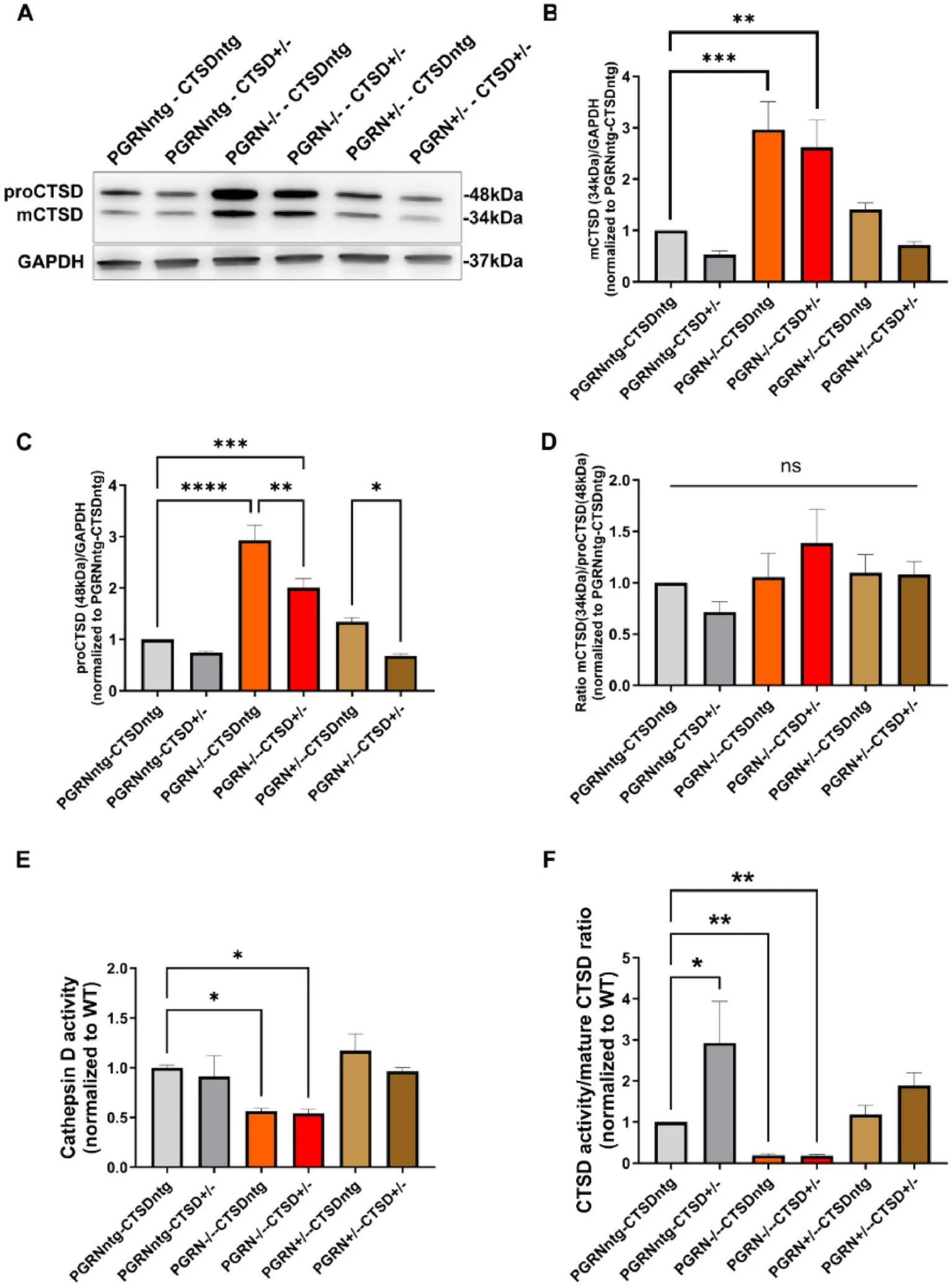
PGRN deficiency results in increased expression of CTSD and reduced CTSD activity in mouse brain tissue. **(A)** Western blot analysis of CTSD protein levels performed on brain lysates of 18-month-old mice with various genotypes, including WT (PGRNntg-CTSDntg), heterozygous CTSD single knockout (PGRNntg – CTSD+/−), homozygous PGRN single knockout (PGRN−/− – CTSDntg), heterozygotes of homozygous PGRN and heterozygous CTSD (PGRN−/− – CTSD+/−), heterozygous PGRN single knockout (PGRN+/− – CTSDntg), and heterozygotes of heterozygous PGRN and heterozygous CTSD (PGRN+/− – CTSD+/−) mice. The blot was probed for mature cathepsin D (mCTSD) and immature cathepsin D (proCTSD), with GAPDH used as a loading control. **(B)** Quantification of mCTSD levels, normalized to GAPDH, shows a significant increase in homozygous PGRN single knockout (PGRN−/− – CTSDntg) and heterozygotes of homozygous PGRN and heterozygous CTSD (PGRN−/− – CTSD+/−) mice compared to WT controls. **(C)** Quantification of proCTSD levels, also normalized to GAPDH, revealed elevated levels in homozygous PGRN single knockout (PGRN−/− – CTSDntg) and heterozygotes of homozygous PGRN and heterozygous CTSD (PGRN−/− – CTSD+/−) compared to WT controls. **(D)** The ratio of mCTSD to proCTSD across different genotypes showed no significant differences between PGRN-deficient and WT mice, suggesting that the increase in CTSD levels is not due to a shift in the relative proportions of mature and immature forms. **(E)** Measurement of CTSD enzymatic activity in brain lysates demonstrated a significant reduction in activity in homozygous PGRN single knockout (PGRN−/− – CTSDntg) and heterozygotes of homozygous PGRN and heterozygous CTSD (PGRN−/− – CTSD+/−) mice compared to WT mice. **(F)** The ratio of CTSD activity to total mCTSD expression levels revealed a pronounced decrease in homozygous PGRN single knockout (PGRN−/− – CTSDntg) and heterozygotes of homozygous PGRN and heterozygous CTSD (PGRN−/− – CTSD+/−) mice, indicating impaired CTSD enzymatic function despite increased mCTSD levels. Statistical significance was determined using a 2-way ANOVA with Tukey’s Post-hoc multiple groups comparison. Data are shown as mean ± SEM of pooled data from six biologically independent mice per group (3 males and 3 females). Statistical significance is indicated as ns (not significant), *p < 0.05, **p < 0.01, ***p < 0.001, ****p < 0.001.

In addition, an *in vitro* enzymatic activity assay was performed on cell lysates to probe for CTSD activity levels (Figure 1E). Interestingly, despite increased CTSD protein levels, two-way ANOVA revealed a significant negative main effect of the PGRN genotype, *F*(2, 30) = 14.14, *p* < 0.0001 on CTSD activity. In contrast, the CTSD genotype (*F*(1, 30) = 0.9313, *p* = 0.3422) or the interaction factor between both genotypes (*F*(2, 30) = 0.5186, *p* = 0.6006) did not significantly impact CTSD activity levels. In addition, *post hoc* multiple group analyses revealed a significant reduction in CTSD activity in homozygous PGRN-deficient mice (PGRN−/− – CTSDntg and PGRN−/− – CTSD+/−), compared to WT controls (*p* = 0.0346 and *p* = 0.0496 respectively) (Figure 1E). This decrease was not attributable to reduced CTSD abundance, as both mature and immature forms were elevated (Figures 1B,C). Notably, after normalization of CTSD enzyme activity to mature CTSD levels the main effect of PGRN genotype remained significant (*F*(2, 30) = 8.927, *p* = 0.0009). *Post hoc* multiple group analysis revealed that this effect was mainly driven by the significant decrease in activity in homozygous PGRN-deficient mice (PGRN−/− – CTSDntg and PGRN−/− – CTSD+/−) compared to WT controls (*p* = 0.0082 and *p* = 0.0076 respectively), whereas no significant difference was found in the heterozygous PGRN-deficient conditions (PGRN+/− – CTSDntg and PGRN+/− – CTSD+/−) (Figure 1F). Surprisingly, upon normalization to mature CTSD levels, the relative activity of CTSD is found to be significantly increased in heterozygous CTSD single knockout (PGRNntg – CTSD+/−) mice (*p* = 0.0431) (Figure 1F). Together, these findings demonstrate that PGRN deficiency leads to increased CTSD expression but impaired functional CTSD enzymatic activity *in vitro*.

### PGRN deficiency increases LAMP1/2 expression and induces perinuclear clustering of LAMP1-positive lysosomes

To further assess the impact of PGRN deficiency on lysosomal function, we investigated the expression of LAMP1/2 and the distribution of LAMP1-positive lysosomes in brain tissue across multiple genotypes. LAMP1 and LAMP2 are well-established canonical structural markers of late endosomal/lysosomal compartments and are frequently used as an indicator of endolysosomal abundance and localization. LAMP1 is primarily considered a structural marker of lysosomes, whereas LAMP2 is additionally involved in lysosomal trafficking and autophagy. We compared LAMP1 and LAMP2 expression in brain lysates from all different generated transgenic mice by Western blot analysis and revealed a significant impact of both PGRN (*F*(2, 30) = 5.938, *p* = 0.0048) and CTSD (*F*(1, 30) = 6.009 *p* = 0.0301) genotypes on LAMP1 protein levels (Figures 2A,B). In contrast, the interaction factor between both genotypes (*F*(2, 30) = 3.019, *p* = 0.2804) did not significantly impact LAMP1 protein levels. Moreover, *post hoc* multiple group analysis revealed increases LAMP1 protein levels in homozygous PGRN-deficient mice (PGRN−/− – CTSDntg; *p* = 0.0081 and PGRN−/− – CTSD+/−; *p* = 0.0041) and heterozygous CTSD single knockout (PGRNntg – CTSD+/−; *p* = 0.0399), compared to WT controls (Figures 2A,B). This elevation suggests either enhanced lysosomal biogenesis or impaired lysosomal turnover in the absence of PGRN. In contrast, LAMP1 levels in the heterozygous PGRN-deficient mice (PGRN+/− – CTSDntg and PGRN+/− – CTSD+/−) were not significantly different from WT, suggesting that complete loss of PGRN is required to elicit this effect. In addition, we also compared LAMP2 expression from all different generated transgenic mice and revealed a similar significant impact of both PGRN (*F*(2, 30) = 11.58, *p* = 0.002) and CTSD (*F*(1, 30) = 6.238 *p* = 0.0178) genotypes on LAMP2 protein levels (Figure 2A; Supplementary Figure S1A). In contrast, the interaction factor between both genotypes (*F*(2, 30) = 2.019, *p* = 0.2943) did not significantly impact LAMP2 protein levels. Moreover, *post hoc* multiple group analysis revealed increases LAMP2 protein levels in homozygous PGRN-deficient mice (PGRN−/− – CTSDntg; *p* = 0.0456 and PGRN−/− – CTSD+/−; *p* = 0.0059) and heterozygous CTSD single knockout (PGRNntg – CTSD+/−; *p* = 0.0433), compared to WT controls (Figure 2A; Supplementary Figure S1A). This elevation suggests either enhanced lysosomal biogenesis or impaired lysosomal turnover in the absence of PGRN. In contrast, LAMP2 levels in the heterozygous PGRN-deficient mice (PGRN+/− – CTSDntg and PGRN+/− – CTSD+/−) were not significantly different from WT, suggesting that complete loss of PGRN is required to elicit this effect. Interestingly, when we calculated the LAMP2/LAMP1 ratio (Supplementary Figure S1B). No significant differences were observed between groups, indicating that the relative balance between these lysosomal membrane markers remained unchanged despite the observed alterations in lysosomal biology.

**Figure 2:**
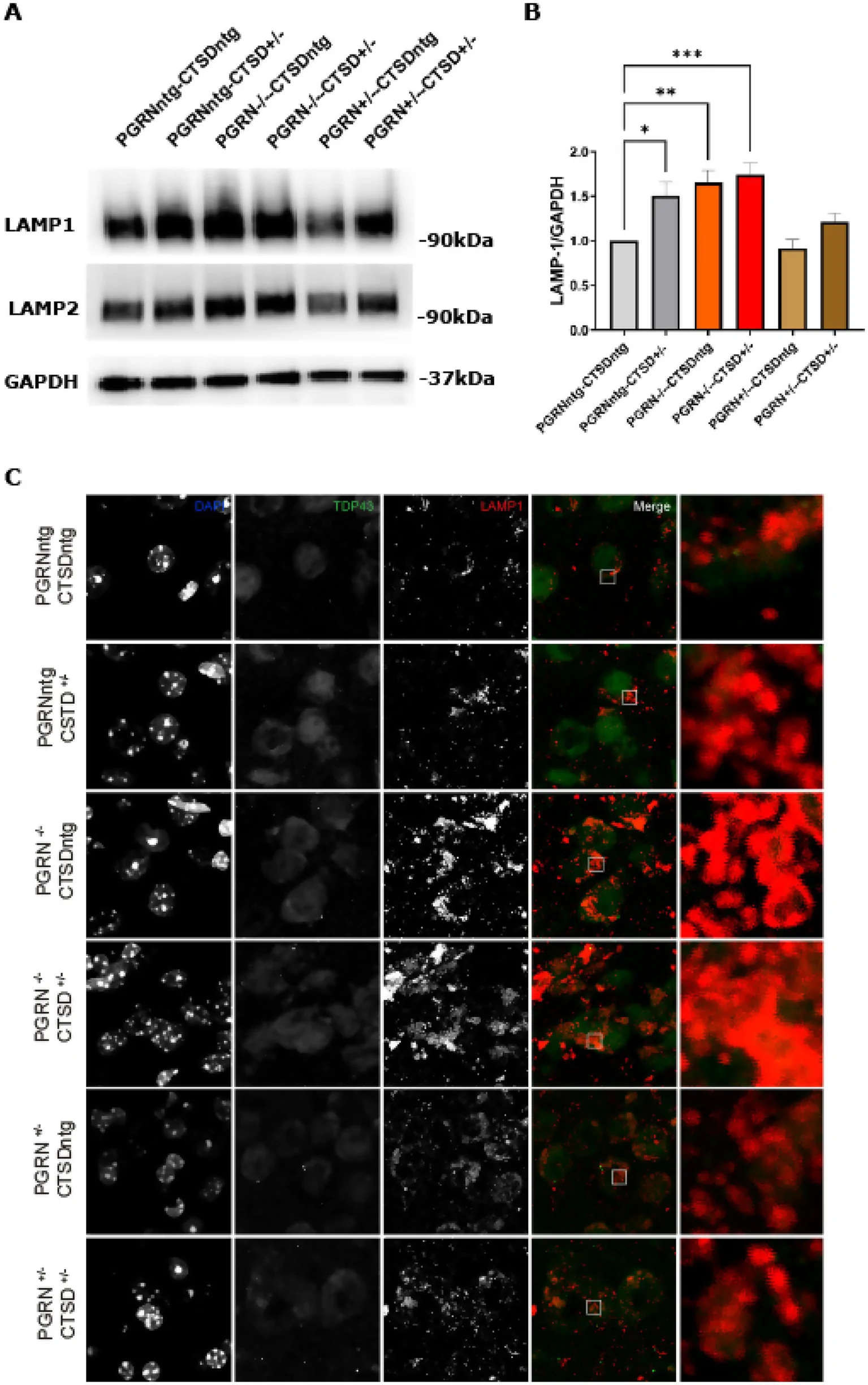
PGRN deficiency results in enhanced levels of LAMP1 proteins and accumulation of LAMP1-positive lysosomes around perinuclear regions. **(A)** Western blots of LAMP1, LAMP2 and GAPDH protein levels of brain lysates from 18-month-old mice in WT (PGRNntg – CTSDntg), heterozygous CTSD single knockout (PGRNntg – CTSD+/−), homozygous PGRN single knockout (PGRN−/− – CTSDntg), heterozygotes of homozygous PGRN and heterozygous CTSD (PGRN−/− – CTSD+/−), heterozygous PGRN single knockout (PGRN+/− – CTSDntg), and heterozygotes of heterozygous PGRN and heterozygous CTSD (PGRN+/− – CTSD+/−) mice. **(B)** Quantification of LAMP1 protein levels normalized to the housekeeping gene and loading control GAPDH. **(C)** Representative immunofluorescent images of brain slices from all abovementioned genotypes revealing an accumulation of LAMP1-positive lysosomes around perinuclear regions in (PGRN−/− – CTSDntg) and (PGRN−/− – CTSD+/−) mice. No significant changes in TDP-43 expression or subcellular localization were observed among the different genotypes. Statistical significance was determined using a 2-way ANOVA with Tukey’s Post-hoc multiple groups comparison. Data are shown as mean ± SEM of pooled data from at least six biologically independent mice per group (equal male:female ratio). Statistical significance is indicated as ns (not significant), *p < 0.05, **p < 0.01, ***p < 0.005.

Immunofluorescence microscopy further demonstrated that LAMP1-positive lysosomes in homozygous PGRN-deficient mice (PGRN−/− – CTSDntg and PGRN−/− – CTSD+/−) were not only increased in abundance and size but also exhibited pronounced perinuclear clustering (Figure 2C). This altered spatial distribution is indicative of defects in lysosomal trafficking, positioning, or fusion dynamics. Owing to the extensive perinuclear clustering of LAMP1-positive structures, accurate segmentation and reliable quantification of their number and size were not feasible. Although image-based quantification was attempted, the resulting measurements were not sufficiently robust and are therefore not presented. Consequently, the immunofluorescence data should be interpreted as qualitative evidence of altered lysosomal homeostasis and spatial organization. While the observed perinuclear clustering is consistent with altered lysosomal organization, the present data do not distinguish whether this phenotype reflects changes in lysosomal trafficking, positioning, fusion dynamics, or compensatory lysosomal accumulation.

Collectively, these findings show that PGRN deficiency disrupts lysosomal abundance and distribution, as evidenced by increased LAMP1/2 expression and perinuclear accumulation of (endo)lysosomes. The observed phenotype further supports a critical role for PGRN in maintaining lysosomal integrity and intracellular trafficking.

### PGRN deficiency alters TDP-43 solubility

To assess the impact of PGRN deficiency on the biochemical properties of TDP-43, we analyzed its distribution in brain tissue using complementary biochemical and immunofluorescence approaches. Brain lysates from the different mouse genotypes were subjected to biochemical fractionation to separate soluble and insoluble fractions, followed by Western blot analysis. In parallel, immunofluorescence microscopy was performed to examine TDP-43 expression and its colocalization with LAMP1-positive lysosomes in brain sections. Western blot analysis revealed no significant changes in TDP-43 levels in either the total or soluble fractions across the different genotypes (Figures 3A–C), indicating that overall TDP-43 abundance and its soluble form are not markedly affected by PGRN deficiency. Similarly, immunofluorescence analysis showed no obvious differences in TDP-43 expression or subcellular localization among the different genotypes (Figure 2C). However, the PGRN genotype did significantly influence TDP-43 protein levels in the insoluble fraction (*F*(2, 36) = 8.052, *p* = 0.0013) in contrast to the CTSD genotype (*F*(1, 36) = 0.3408, *p* = 0.5630) or the interaction factor between both genotypes (*F*(2, 36) = 1.442, *p* = 0.2499) which did not significantly impact insoluble TDP-43 levels (Figures 3D,E). *Post hoc* multiple group analysis revealed significantly increased insoluble TDP-43 levels in homozygous PGRN-deficient mice (PGRN−/− – CTSDntg; *p* = 0.0462 and PGRN−/− – CTSD+/−; *p* = 0.0409), as well as heterozygous PGRN-deficient mice (PGRN+/− – CTSDntg; *p* = 0.0389), compared to WT controls (Figures 3D,E). These findings indicate that PGRN deficiency promotes a shift of TDP-43 towards the detergent-insoluble fraction, suggesting altered TDP-43 biochemical partitioning and potentially impaired protein homeostasis. However, in the absence of detectable phosphorylated TDP-43 cytoplasmic inclusions, these changes should be interpreted as early biochemical alterations rather than evidence of established TDP-43 proteinopathy.

**Figure 3:**
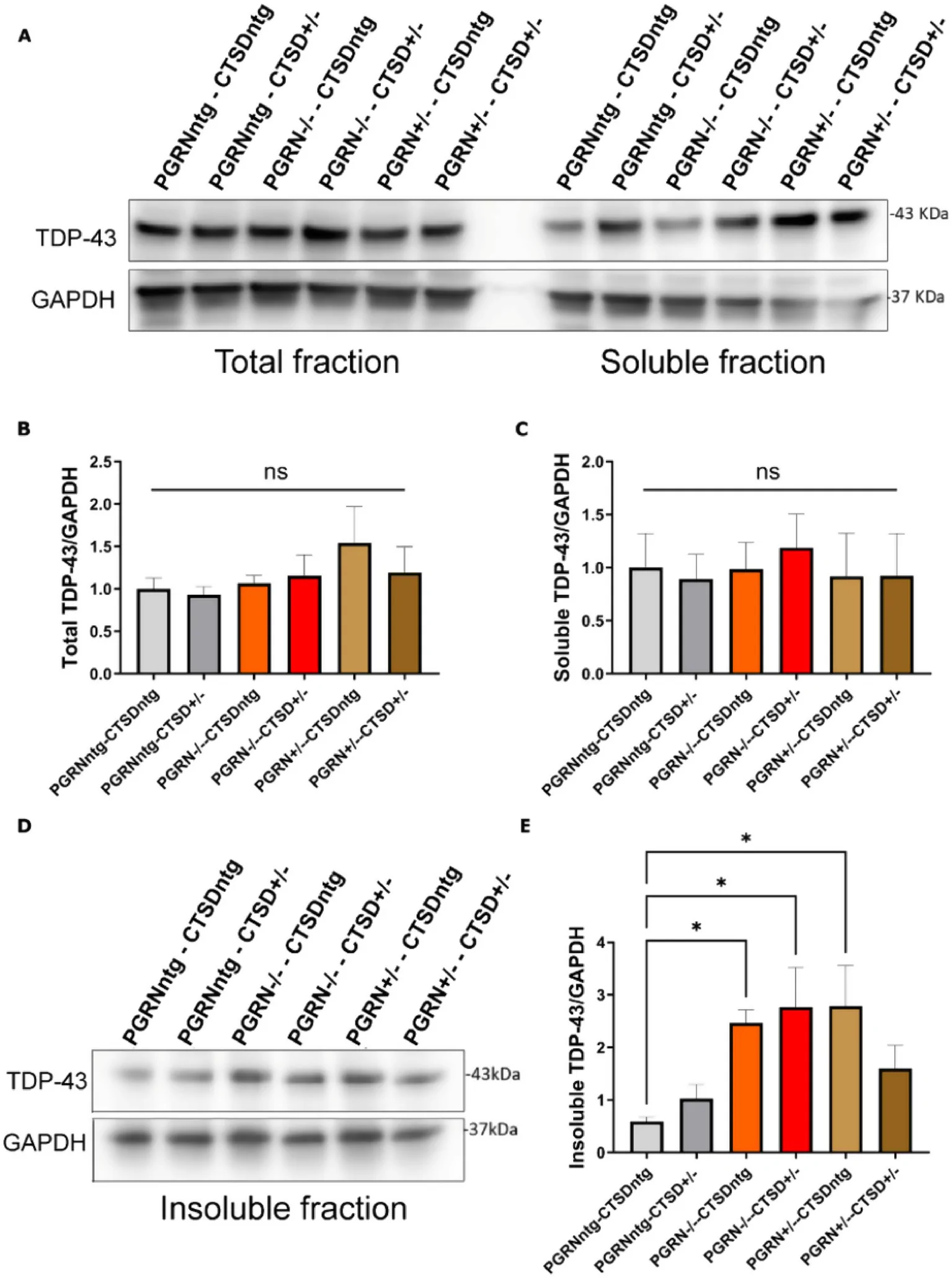
PGRN deficiency increases insoluble TDP-43 levels. **(A)** Western blot analysis of brain lysates from 18-month-old mice with various genotypes, including WT (PGRNntg – CTSDntg), heterozygous CTSD single knockout (PGRNntg – CTSD+/−), homozygous PGRN single knockout (PGRN−/− – CTSDntg), heterozygotes of homozygous PGRN and heterozygous CTSD (PGRN−/− – CTSD+/−), heterozygous PGRN single knockout (PGRN+/− – CTSDntg), and heterozygotes of heterozygous PGRN and heterozygous CTSD (PGRN+/− – CTSD+/−) mice. The blot was probed for TDP-43, with GAPDH serving as a loading control. The analysis includes both total and insoluble TDP-43 fractions. **(B)** Quantification of total TDP-43 levels from the Western blot analysis, normalized to GAPDH, showed no significant differences between genotypes and WT controls. **(C)** Quantification of soluble TDP-43 levels, normalized to GAPDH, showed no significant differences between genotypes and WT controls. **(D)** Western blot analysis of insoluble TDP-43 in brain lysates from WT, homozygous PGRN single knockout (PGRN−/− – CTSDntg), and heterozygotes of homozygous PGRN and heterozygous CTSD (PGRN−/− – CTSD+/−) mice, with GAPDH used as a loading control. **(E)** Quantification of insoluble TDP-43 levels revealed a significant increase in homozygous PGRN single knockout (PGRN−/− – CTSDntg) and in heterozygotes of homozygous PGRN and heterozygous CTSD (PGRN−/− – CTSD+/−) mice compared to WT controls, indicating a buildup of insoluble TDP-43 associated with PGRN deficiency. Statistical significance was determined using a 2-way ANOVA with Tukey’s Post-hoc multiple groups comparison. Data are presented as mean ± SEM. For Figure 1B, pooled data from *n* = 12 biologically independent mice per group (6 males and 6 females) are shown. For Figures 1C,E, pooled data from *n* = 6 biologically independent mice per group (3 males and 3 females) are shown.

However, to determine whether PRGN or CTSD deficiency resulted in overt pathological TDP-43 abnormalities, whole-brain, coronal sections from end-stage mice were immunostained and inspected for the presence of cytoplasmic phosphorylated-TDP43 inclusions (Supplementary Figure S2). Specifically, regions of interest usually affected by pTDP-43 pathology in frontotemporal lobar degeneration individuals (temporal cortex) were imaged. No detectable cytoplasmic TDP-43 aggregates were observed in neurons or glial cells in either of the study groups at the analyzed timepoint. Although PRGN mutations have been associated with TDP-43 pathology in human disease, our findings suggest that the loss of these genes alone (or in combination) does not lead to abnormal TDP-43 distribution.

Collectively, these results demonstrate that PGRN deficiency selectively promotes the accumulation of insoluble TDP-43 without altering total protein levels, highlighting a critical role for PGRN in maintaining protein homeostasis and suggesting a potential upstream contribution to TDP-43 proteinopathy.

### Double transgenic, homozygous PGRN and heterozygous CTSD mice have shorter lifespans

Although lifespan and mortality are not used as primary endpoint in (PGRN) FTD models, we did analyze the lifespan of our different heterozygous and homozygous transgenic mice in order to provide a comprehensive assessment of genotype-dependent consequences on long-term organismal health (Figures 4A,B). Kaplan–Meier survival analysis revealed significant differences in survival among the six experimental groups (log-rank [Mantel–Cox] test, χ^2^(5) = 113.7, *p* < 0.0001, median survival ± 95% CI shown in figure legend). In fact, we noticed a marked reduction in lifespan of homozygous PGRN and heterozygous CTSD (PGRN−/− – CTSD+/−) mice compared to all other genotypes. Specifically, PGRN−/− – CTSD+/− mice exhibited a median survival of only 639 days, with many animals failing to reach advanced age. In contrast, WT, PGRN−/− – CTSDntg, and PGRNntg – CTSD+/− mice displayed normal or only modestly reduced lifespans (Figures 4A,B), indicating that neither PGRN deficiency nor partial CTSD loss alone is sufficient to produce this pronounced survival phenotype.

**Figure 4:**
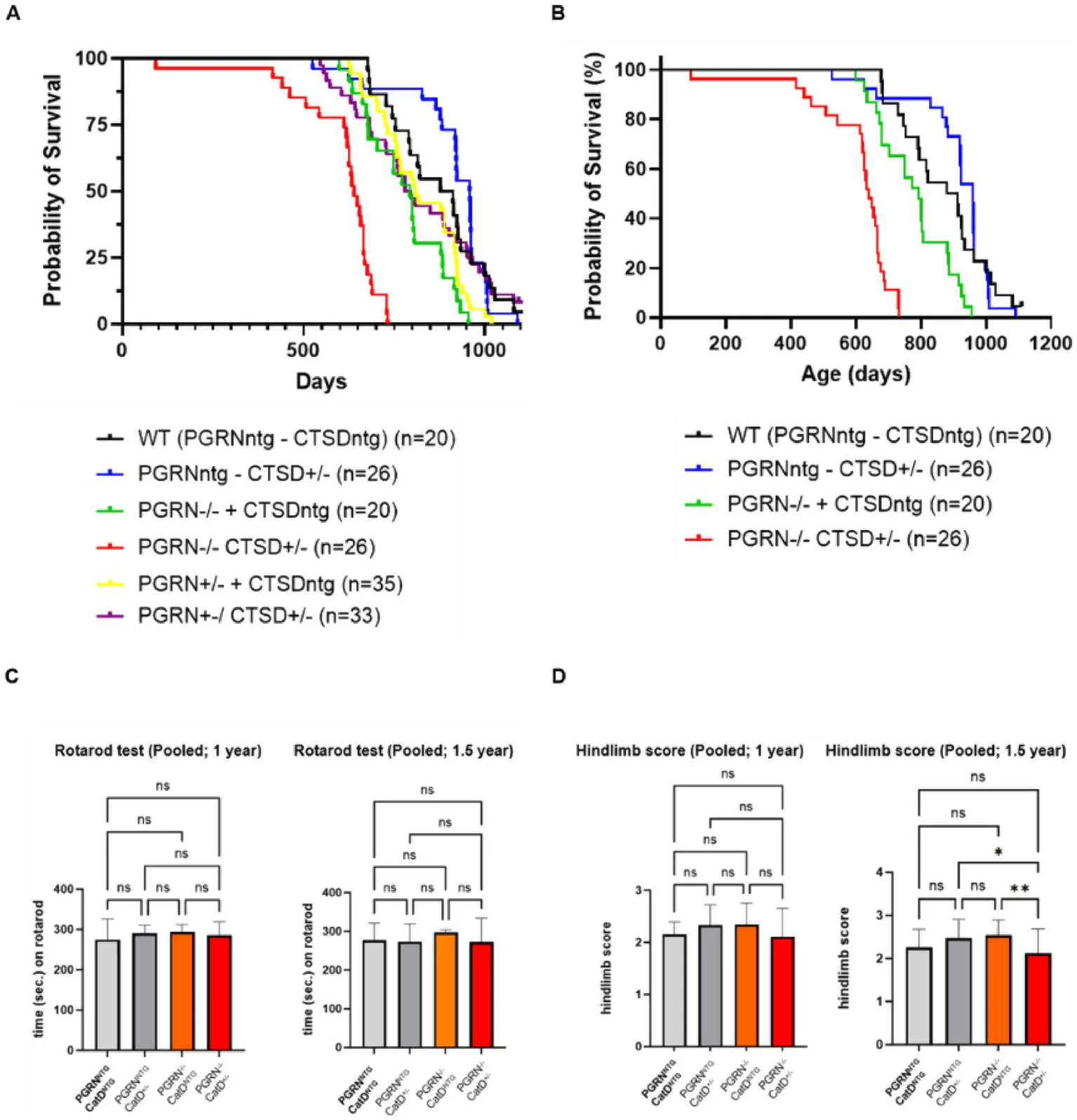
PGRN deficiency does not markedly affect lifespan although it shows signs of motor dysfunctions. **(A)** Kaplan–Meier survival curves showing the survival of WT (PGRNntg – CTSDntg), heterozygous CTSD single knockout (PGRNntg – CTSD+/−), homozygous PGRN single knockout (PGRN−/− – CTSDntg), heterozygotes of homozygous PGRN and heterozygous CTSD (PGRN−/− – CTSD+/−), heterozygous PGRN single knockout (PGRN+/− – CTSDntg), and heterozygotes of heterozygous PGRN and heterozygous CTSD (PGRN+/− – CTSD+/−) mice. Survival was analyzed using the log-rank (Mantel–Cox) test. Sample sizes and median survival were WT (*n* = 20; 895 days; 95% CI 753−934), PGRNntg – CTSD+/− (*n* = 26; 950 days; 95% CI 908−956), PGRN−/− – CTSDntg (*n* = 20; 793 days; 95% CI 679–805), PGRN−/− – CTSD+/− (*n* = 26; 639 days; 95% CI 618−666), PGRN+/− – CTSDntg (*n* = 35; 808 days; 95% CI 757−888), and PGRN+/− – CTSD+/− (*n* = 33; 788 days; 95% CI 727−902). **(B)** Detailed survival curves comparing WT, heterozygous CTSD single knockout (PGRNntg – CTSD+/−), homozygous PGRN single knockout (PGRN−/− – CTSDntg) and heterozygotes of homozygous PGRN and heterozygous CTSD (PGRN−/− – CTSD+/−) mice. This panel provides a closer look at the survival differences, indicating that while PGRN deficiency does not drastically reduce lifespan, it may influence longevity in specific contexts. **(C)** Motor coordination and balance were assessed using the rotarod test at 12 and 18 months of age. Data are presented as mean ± SEM. Sample sizes were WT (*n* = 10), CTSD+/− (*n* = 38), PGRN−/− (*n* = 30), and PGRN−/− – CTSD+/− (*n* = 27). **(D)** Hindlimb clasping scores were determined at 12 and 18 months of age. PGRN−/− – CTSD+/− mice displayed significantly impaired hindlimb function compared with PGRN−/− mice at 18 months. Survival differences were evaluated using a log-rank (Mantel–Cox) test (χ^2^ = 113.7, df = 5, *p* < 0.0001). Statistical significance for behavioral analyses was determined using one-way ANOVA with Tukey’s Post-hoc multiple groups comparison. Data are presented as mean ± SEM. Sample sizes were WT (*n* = 10), CTSD+/− (*n* = 38), PGRN−/− (*n* = 27), and PGRN−/−; CTSD+/− (*n* = 21). Statistical significance is indicated as ns (not significant), *p < 0.05 and **p < 0.01.

To further assess the functional consequences of these genetic alterations, we evaluated motor performance and coordination in mice at 12 and 18 months of age, as these timepoints are widely employed in FTD mouse models for the assessment of disease-relevant behavioral deficits. Rotarod testing at 12 and 18 months of age showed no significant differences between genotypes. However, hindlimb clasping scores at 18 months revealed a significant impairment in PGRN−/− – CTSD+/− mice when compared to both PGRNntg – CTSD+/− (*p* = 0.0222) and PGRN−/− – CTSDntg mice (*p* = 0.009) (Figures 4C,D), suggesting emerging neurological deficits in the combined knockout condition. Together, these findings indicate a synergistic interaction between PGRN deficiency and reduced CTSD levels, leading to decreased survival and late-onset motor impairment. This supports a critical cooperative role for PGRN and CTSD in maintaining neuronal function and organismal longevity, likely through preservation of lysosomal integrity and cellular homeostasis.

### Heterozygous CTSD loss increases progranulin levels and modulates CTSD activity

To evaluate whether CTSD influences PGRN levels, we quantified PGRN in brain lysates from different genotypes using an enzyme-linked immunosorbent assay (ELISA). As expected, PGRN levels were significantly reduced in all PGRN-deficient conditions, including homozygous PGRN single knockout and double knockout of homozygous PGRN and heterozygous CTSD (PGRN−/− – CTSDntg and PGRN−/− – CTSD+/−) where PGRN protein levels were undetectable (Figure 5A).

**Figure 5:**
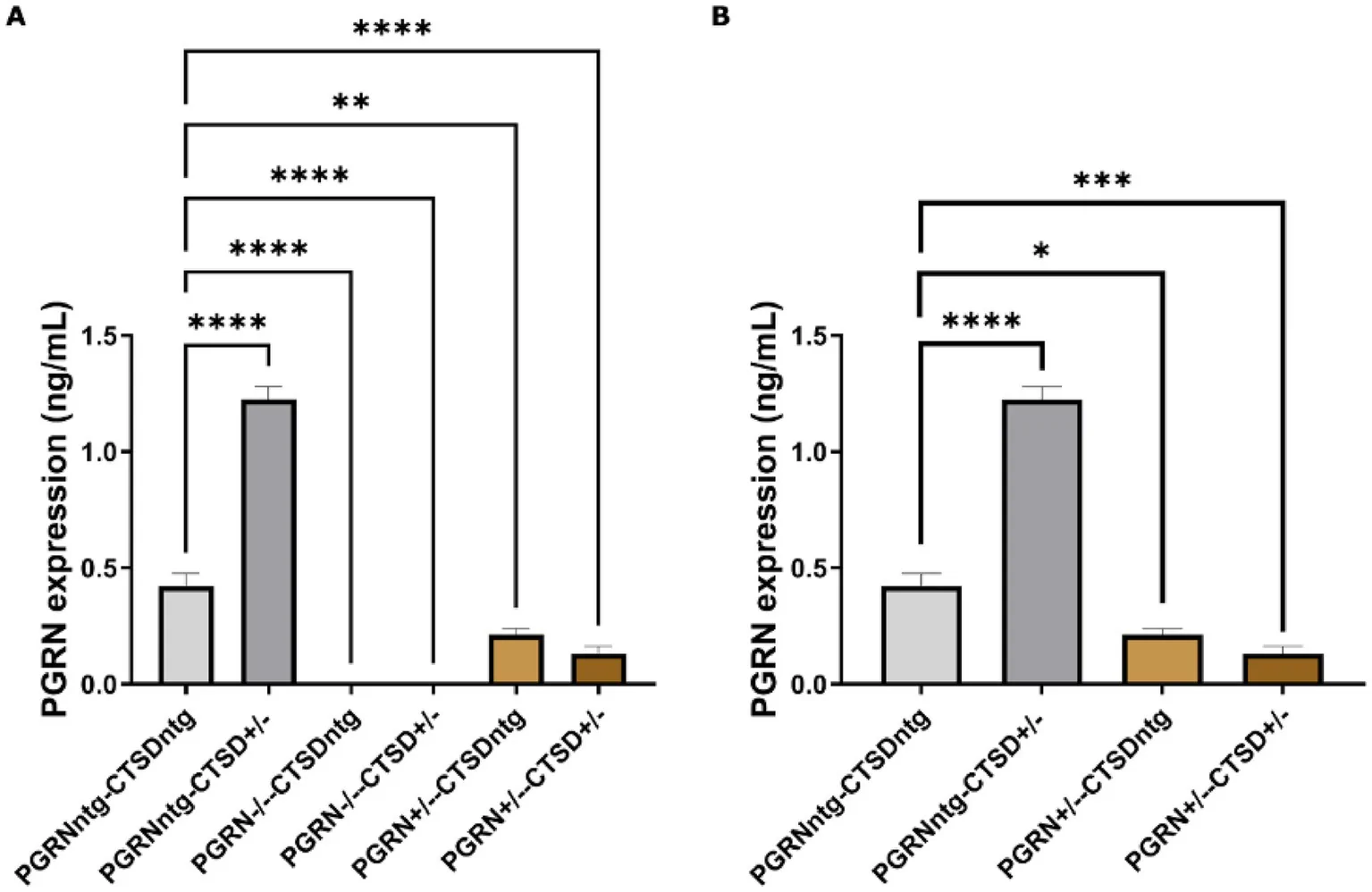
Reduction in CTSD levels influences progranulin levels. **(A)** PGRN levels in brain lysates from 18-month old mice measured by ELISA in different mouse genotypes, including WT (PGRNntg – CTSDntg), heterozygous single knockout (PGRNntg – CTSD+/−), homozygous PGRN single knockout (PGRN−/− – CTSDntg), double knockout of homozygous PGRN and heterozygous CTSD (PGRN−/− – CTSD+/−), heterozygous PGRN single knockout (PGRN+/− – CTSDntg) and double knockout heterozygous PGRN and heterozygous CTSD (PGRN+/− – CTSD+/−) mice. **(B)** Representation and analysis of the data presented in **(A)** excluding the homozygous PGRN knockout mice. Statistical significance was determined using a 2-way ANOVA with Tukey’s Post-hoc multiple groups comparison. Data are shown as mean ± SEM of pooled data from six biologically independent mice per group (3 males and 3 females). Statistical significance is indicated as ns (not significant), *p < 0.05, **p < 0.01,***p < 0.001,****p < 0.0001.

However, because the absence of PGRN expression in these animals is an intrinsic consequence of the genomic PGRN knockout, statistical comparisons of PGRN levels in these genotypes are of limited biological relevance. Therefore, the effect of PGRN and CTSD genotypes on PGRN protein abundance was evaluated within PGRN-expressing animals only (Figure 5B). In line with our expectations, a significant main effect of both PGRN (*F*(1, 20) = 216.6, *p* < 0.0001) and CTSD (*F*(1, 20) = 66.58 *p* < 0.0001) genotypes was observed. On top of that, the interaction factor between both genotypes (*F*(1, 20) = 100.9, *p* < 0.0001) also significantly impacted PGRN protein levels. As expected, multiple group analysis revealed that heterozygous PGRN knockout significantly reduced PGRN levels (PGRN+/− – CTSDntg; *p* = 0.0171 and PGRN+/− – CTSD+/−; *p* = 0.0008) (Figure 5B). In contrast, a significant increase in PGRN levels was observed in heterozygous CTSD single knockout (PGRNntg – CTSD+/−) mice compared to controls (Figure 5B). In addition, because PGRN levels have been reported to show sex-dependent regulation in some contexts (Houser et al., 2022; Nicholson et al., 2014; Zhang et al., 2023), we performed an additional *post hoc* analysis of the ELISA dataset where male and female mice were analyzed separately (Supplementary Figures 3A,B). This analysis did not reveal any major sex-dependent differences across genotypes that altered the interpretation of the findings. Collectively, these results support a functional interplay between PGRN and CTSD, highlighting a potential feedback mechanism that may regulate lysosomal protease activity and total brain PGRN levels.

## Discussion

FTD is a progressive neurodegenerative disorder, marked by early-onset cognitive decline and characterized by selective neuronal loss in the frontal and temporal lobes (Grossman et al., 2023). Despite strong genetic associations with mutations in *MAPT*, *GRN*, and *C9orf72* (Greaves and Rohrer, 2019), the underlying mechanisms of FTD remain poorly understood, limiting the development of effective therapeutic interventions. In this study, we investigated the role of PGRN in FTD, particularly its interactions with CTSD and lysosomal function. Using mouse models with various combinations of PGRN and CTSD deficiencies, we identified molecular and cellular alterations linking PGRN deficiency to lysosomal dysfunction and altered TDP-43 homeostasis (Figure 6).

**Figure 6:**
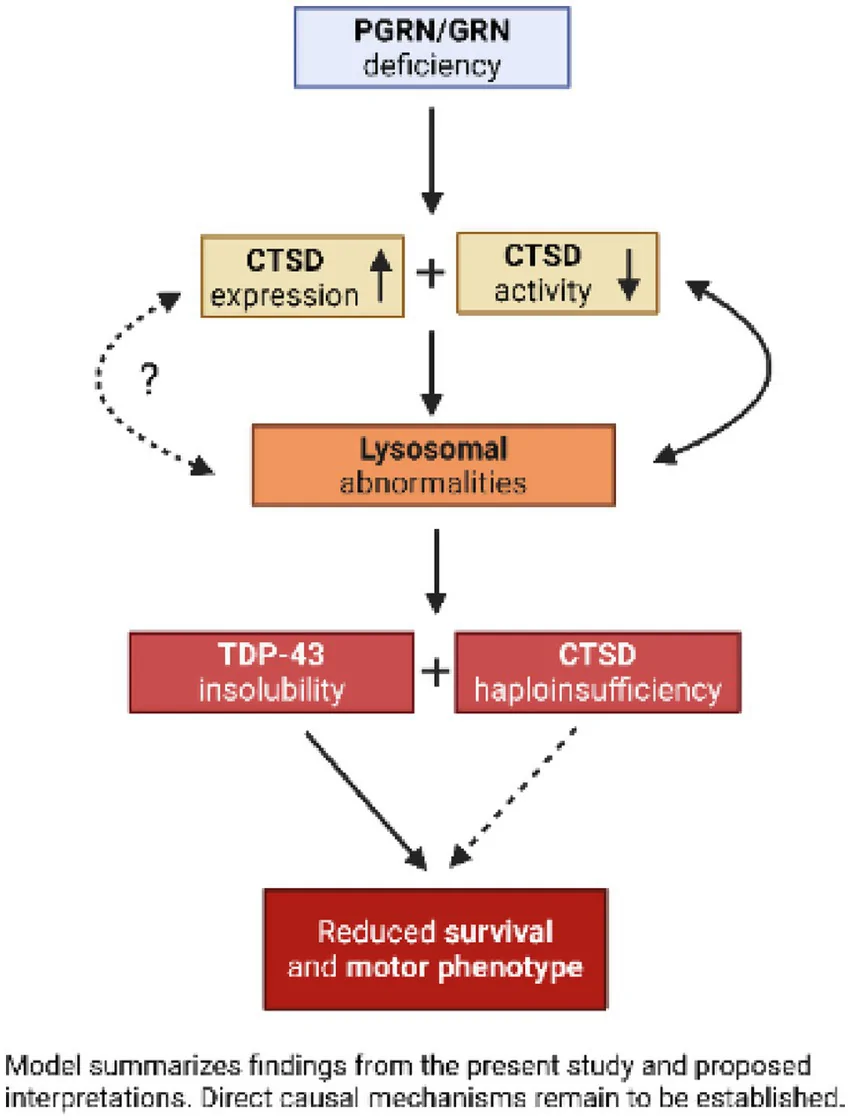
Schematic representation of the proposed role of CTSD as a modifier of PGRN-associated disease.

Our analyses revealed that PGRN deficiency leads to increased levels of both mature (mCTSD) and immature (proCTSD) CTSD in homozygous PGRN single knockout (PGRN−/− – CTSDntg) and double knockout of homozygous PGRN and heterozygous CTSD (PGRN−/− – CTSD+/−) mice, compared to WT controls. While we did not observe any marked alterations in the processing of CTSD given that the ratio of mature-to-immature CTSD remained unchanged, this measurement alone does not exclude potential differences in processing flux, enzymatic turnover, or other aspects of CTSD maturation and regulation that still need to be investigated.

Surprisingly, despite the observed CTSD elevation in homozygous PGRN knockout mice, using an *in vitro* assay on cell lysates, CTSD enzymatic activity was found to be significantly reduced. This paradoxical finding suggests that PGRN is not required for CTSD expression per se, but is critical for its proper enzymatic activity, indicating a functional uncoupling between CTSD abundance and activity that is consistent with the proposed role of PGRN as a chaperone for CTSD (Beel et al., 2018). Because neither mature nor immature CTSD levels are reduced, the observed loss of enzymatic activity relative to mature CTSD levels likely reflects defects in CTSD maturation, processing, subcellular localization, activation state, or the proportion of mature CTSD that is enzymatically active rather than altered protein expression. These findings align with the lysosomal dysfunction reported in PGRN-deficient models and underscore a complex regulatory role for PGRN in maintaining lysosomal protease activity and overall lysosomal homeostasis.

Our investigation of lysosomal markers showed increased LAMP1 levels and altered lysosomal distribution in homozygous PGRN single knockout (PGRN−/− – CTSDntg) and double knockout of homozygous PGRN and heterozygous CTSD (PGRN−/− – CTSD+/−) mice. The elevated LAMP1 levels suggest that PGRN deficiency disrupts lysosomal homeostasis or induces compensatory lysosomal biogenesis. In addition, the unchanged LAMP2/LAMP1 ratio suggests that the observed lysosomal alterations are unlikely to result from major changes in lysosomal maturation or function, at least as reflected by the relative abundance of these two lysosomal membrane proteins. The perinuclear accumulation of LAMP1-positive lysosomes points to impaired lysosomal trafficking or fusion, which could further exacerbate lysosomal storage disorders. Together, these data indicate that PGRN deficiency disrupts both lysosomal function and organization.

PGRN is essential for proper localization and function of TDP-43, a protein commonly aggregated in FTD. In fact, previous studies have shown that PGRN deficiency can lead to significant cytoplasmic TDP-43 accumulation and aggregation, and that its deficiency promotes cytoplasmic accumulation and aggregation (Chang et al., 2017; Kumar-Singh, 2011). Fractionation of brain mouse brain lysates revealed increased levels of TDP-43 in the insoluble fraction of homozygous PGRN single knockout (PGRN−/− – CTSDntg), double knockout of homozygous PGRN and heterozygous CTSD (PGRN−/− – CTSD+/−) and heterozygous PGRN single knockout (PGRN+/− – CTSDntg) mice. However, since canonical TDP-43 pathology in FTD is defined by hyperphosphorylation, cytoplasmic mislocalization, and/or inclusion formation of TDP-43, the inability to detect these pathological hallmarks in our mice represents an important limitation of this study. Nevertheless, this accumulation of TDP-43 in the insoluble protein fraction suggests that biochemical accumulation may precede detectable histopathological aggregation. In fact, most studies using similar homozygous PGRN knockout mice also mention the lack of overt TDP-43 pathology (Ahmed et al., 2010; Ghoshal et al., 2012; Yin et al., 2010). The observed increase in insoluble TDP-43 is likely attributable to lysosomal dysfunction caused by PGRN deficiency, which compromises the autophagic clearance of misfolded or aggregated proteins such as TDP-43 (Kumar et al., 2021; Scotter et al., 2014). Taken together, the enhanced insoluble TDP-43 levels observed in this study underscore the role of PGRN in maintaining protein homeostasis and preventing TDP-43 aggregation, a critical aspect of FTD pathology. This is in line with a previous study from our lab in which the accumulation of insoluble TDP-43 in mutant TARDBP mice was reduced by overexpression of PGRN (Beel et al., 2018). Therefore, while our findings do not directly demonstrate overt disease-relevant TDP-43 pathology or neurodegeneration, they are consistent with early proteostatic disturbances that may predispose to FTD-related neurodegeneration. These processes may represent biologically relevant upstream events requiring further investigation in models with more robust neurodegenerative phenotypes.

Consistent with these molecular and cellular phenotypes, lifespan analysis revealed that heterozygotes of homozygous PGRN and heterozygous CTSD (PGRN−/− – CTSD+/−) mice exhibit significantly reduced survival compared to other genotypes, highlighting a synergistic effect of PGRN deficiency and partial CTSD loss. These findings raise the possibility that CTSD functions as a genetic or functional modifier of GRN-associated disease, whereby reduced CTSD levels exacerbate the consequences of progranulin deficiency without being sufficient to independently drive disease pathology. Interestingly, biochemical and cellular abnormalities associated with progranulin deficiency were already evident at earlier ages, well before any detectable differences in survival emerged, indicating that these biochemical alterations precede overt effects on lifespan and likely contribute to the subsequent decline in organismal health.

Finally, the lack of overt survival phenotypes in heterozygous PGRN mice (PGRN+/−) and the modest (non-significant) reduction in survival in homozygous PGRN mice (PGRN−/−) is consistent with prior studies using similar PGRN-FTD mice models (Ghoshal et al., 2012; Nguyen et al., 2018).

Notably, PGRN levels were reduced in both homozygous and heterozygous knockout conditions, while heterozygous CTSD loss led to elevated PGRN levels. This increase in PGRN levels may be linked to the compensatory mechanisms associated with altered CTSD activity, as increased CTSD activity was observed in this genotype during our initial experiments. These findings suggest a bidirectional regulatory relationship between PGRN and CTSD, in which CTSD levels may influence PGRN abundance, while PGRN, in turn, modulates CTSD activity.

### Limitations and future perspectives

Several limitations should be considered when interpreting CTSD-related findings. Although the ratio of mature CTSD to proCTSD was unchanged between experimental groups, suggesting that major alterations in CTSD processing were not evident under the conditions studied, this measure alone cannot exclude potential differences in processing flux, protein turnover, trafficking, or other regulatory aspects of CTSD maturation. In fact, previous studies have demonstrated that CTSD maturation is a multi-step process regulated by several proteolytic and lysosomal pathways (Laurent-Matha et al., 2006; Mijanovic et al., 2021). Consequently, subtle changes in CTSD biogenesis or trafficking may not be captured by the steady-state measurements of pro- and mature CTSD abundance performed here.

In addition, while normalization of CTSD activity to mature CTSD protein levels revealed reduced activity relative to mature enzyme abundance *in vitro*, these data should be interpreted with caution. The assay provides a measure of overall functional activity in cell lysates but does not allow discrimination between distinct mechanisms that could contribute to reduced activity. Such mechanisms may include changes in catalytic efficiency, intracellular localization, lysosomal microenvironment, enzyme activation state, or the proportion of mature CTSD that is enzymatically active (Mijanovic et al., 2021; Yadati et al., 2020). Importantly, because CTSD activity was assessed *in vitro* rather than within intact lysosomes, the observed reduction reflects decreased catalytic capacity under the assay conditions and may not fully represent physiological lysosomal proteolytic activity or degradative function in living cells. Therefore, our findings should be considered consistent with reduced CTSD catalytic activity measured *in vitro* relative to mature protein levels rather than definitive evidence of impaired CTSD function or lysosomal degradation *in vivo* through a specific mechanism. Future studies employing complementary approaches that assess lysosomal function in living cells would provide important additional insight into the physiological consequences of progranulin deficiency. Such approaches may include DQ-BSA degradation assays, Magic Red CTSD activity measurements, LysoTracker-based analyses of lysosomal abundance and integrity, and direct assessment of lysosomal pH. These methods would help determine whether the reduced catalytic capacity observed in cell lysates translates into altered lysosomal function under physiological conditions.

Moreover, although our mechanistic interpretation of dysfunctional lysosomal proteolysis focuses on CTSD, it is important to recognize that PGRN functions within a broader lysosomal protease network. Several studies have demonstrated a specific functional relationship between PGRN and CTSD, including direct interaction and enhancement of CTSD enzymatic activity by PGRN, supporting a role for CTSD as a biologically relevant downstream effector of PGRN-dependent lysosomal function (Beel et al., 2018; Valdez et al., 2017). However, PGRN metabolism is not restricted to CTSD. Intralysosomal processing of PGRN into granulin peptides involves multiple proteases, including the cysteine cathepsins cathepsin L (CTSL) and cathepsin B (CTSB), as well as several additional lysosomal enzymes (Mohan et al., 2021; Zhou et al., 2017a). CTSL has been shown to efficiently cleave PGRN, and systematic analyses indicate that multiple cathepsins contribute to PGRN processing in a protease- and pH-dependent manner (Mohan et al., 2021; Zhou et al., 2017a). Therefore, changes in PGRN expression or function may affect several interconnected lysosomal proteolytic pathways rather than a single cathepsin. Consequently, although our findings are consistent with a CTSD-related mechanism, the present study did not directly evaluate the activity or expression of other lysosomal proteases such as CTSB or CTSL. We therefore cannot exclude the possibility that the observed phenotypes reflect broader alterations in lysosomal protease homeostasis. Future studies incorporating parallel assessment of cysteine and aspartyl cathepsin pathways will be important for establishing the degree of CTSD specificity and for defining how PGRN coordinates the wider lysosomal degradative network.

An obvious limitation of the present study is that the transgenic mouse models that were used to investigate the relationship between PGRN deficiency and CTSD-mediated lysosomal dysfunction did not develop robust neurodegenerative phenotypes, marked behavioral impairment, or substantial survival deficits. While pathogenic variants in GRN are a major genetic cause of FTD in humans, the relatively mild phenotype observed in our model is consistent with a well-recognized limitation of many PGRN-deficient mouse models. Previous studies have shown that PGRN deficiency in mice results in disease-relevant lysosomal, inflammatory, and neurodegenerative alterations, yet often fails to fully recapitulate the severity of human FTD. For example, aged PGRN−/− mice develop lipofuscinosis, gliosis, and lysosomal dysfunction without overt FTLD-U pathology or pronounced neurodegeneration (Ahmed et al., 2010), while other studies have reported neuroinflammatory and behavioral abnormalities but only limited neuropathological manifestations (Yin et al., 2010). More recently, Yang and colleagues demonstrated that phenotypic severity is highly influenced by genetic background and highlighted that the commonly used C57BL/6-based PGRN-deficient models generally display comparatively mild disease manifestations (Yang et al., 2025).

The behavioral characterization of the mouse models presented in this manuscript is limited. While multiple behavioral paradigms, including cognitive, anxiety-related, social, and spontaneous behavioral assessments, were evaluated during the characterization of PGRN-deficient mice, no robust or reproducible genotype-dependent abnormalities were detected. In particular, cognitive testing and social interaction paradigms did not reveal significant deficits compared with wild-type controls. Consequently, the present study focused on motor function as a measurable functional outcome. Nevertheless, we acknowledge that more comprehensive behavioral testing at additional ages, particularly in the combined PGRN−/−; CTSD+/− mice, may provide complementary insights and reveal subtle cognitive or behavioral abnormalities that were not captured in the present study.

Surprisingly, but consistent with existing literature, we observed only modest effects on survival. Importantly, lifespan is generally preserved in PGRN+/− mice despite the presence of clear disease-relevant cellular and molecular abnormalities, whereas complete loss of PGRN may reduce survival, although these effects are typically modest, strain-dependent, and inconsistently reported (Ghoshal et al., 2012; Nguyen et al., 2018). Nevertheless, the molecular and biochemical alterations identified in our study were detected well before any measurable effects on survival, suggesting that they reflect chronic, late-stage biochemical adaptations rather than secondary consequences of end-stage disease. This concept is consistent with current views of FTD pathogenesis, in which disease-relevant molecular abnormalities emerge long before overt neurodegeneration becomes apparent. Moreover, although progranulin deficiency has previously been associated with altered inflammatory responses and microglial activation, inflammatory signaling was not directly investigated in the present study. Therefore, we cannot determine whether the observed lysosomal abnormalities are accompanied by changes in pro- or anti-inflammatory pathways. Future studies examining cytokine expression and glial activation will be important to establish how CTSD-dependent lysosomal dysfunction contributes to neuroinflammation in progranulin-deficient models.

Another limitation is the absence of overt TDP-43 proteinopathy. Phosphorylated TDP-43 inclusions, nuclear clearance, and cytoplasmic accumulation are hallmark pathological features of GRN-associated FTD in humans, yet these abnormalities are frequently absent or only minimally detectable in currently available PGRN-deficient mouse models. Indeed, the lack of overt TDP-43 pathology has been repeatedly documented in the literature. Ahmed et al. reported no evidence of FTLD-U-like pathology in aged PGRN−/− animals (Ahmed et al., 2010), while Yin et al. observed only subtle TDP-43 abnormalities despite clear neuroinflammatory and lysosomal phenotypes (Yin et al., 2010). Similarly, Ghoshal et al. (2012) extensively examined 24-month-old PGRN−/− mice and found no evidence of classical TDP-43 proteinopathy, including neuronal cytoplasmic inclusions, neuronal intranuclear inclusions, dystrophic neurites, or glial inclusions, despite clear disease-relevant abnormalities elsewhere in the brain. Consistent with these reports, we did not detect phosphorylated TDP-43 inclusions in our model. Although this represents a limitation with respect to modelling the full neuropathological spectrum of human FTD, it does not invalidate the disease relevance of the observed molecular phenotypes. Instead, our findings suggest that the increase in insoluble TDP-43 is a chronic, late-stage biochemical adaptation and may reflect an early, pre-aggregated state that precedes overt inclusion formation.

Unfortunately, all PGRN measurements in the present study were performed on whole-brain lysates. Consequently, the ELISA data reflect total tissue PGRN levels and do not provide information regarding subcellular localization or lysosome-specific protein abundance. Therefore, while our findings demonstrate changes in overall brain PGRN levels, they do not directly establish whether these alterations occur within lysosomes or other intracellular compartments. We therefore have to interpret these results with caution and cannot exclude contributions from non-lysosomal pools of PGRN. However, the observed findings remain relevant in the context of lysosomal biology because both progranulin and cathepsin D are key lysosomal proteins with well-established functional interactions (Hu et al., 2010; Mohan et al., 2021; Valdez et al., 2017; Zhou et al., 2017a). Moreover, a more direct and detailed assessment of lysosomal localization and morphology was beyond the scope of the current study, and future experiments employing lysosomal fractionation or imaging-based localization approaches will be necessary to determine the compartment-specific basis of the observed changes.

Importantly, the present study was not designed to directly assess the neurodegenerative process underlying frontotemporal dementia. Although we observed lysosomal abnormalities, altered CTSD activity, increased insoluble TDP-43, motor deficits, and reduced survival in specific genotypes, we did not examine neuronal vulnerability or loss, region-specific neurodegeneration, cognitive or social behavioral abnormalities, or inflammatory responses. Future experiments using primary neuronal cultures and/or quantitative histological analyses of vulnerable brain regions will be required to determine whether CTSD deficiency increases neuronal susceptibility or neuronal loss in the context of progranulin deficiency. Consequently, the current findings should be interpreted primarily as evidence that CTSD modulates molecular and cellular phenotypes associated with progranulin deficiency. Whether these abnormalities directly contribute to neurodegeneration and clinical disease progression in GRN-associated FTD remains to be determined.

## Concluding remarks and future perspectives

Accumulating evidence indicates that lysosomal dysfunction, altered proteostasis, and disturbances in TDP-43 biology can occur in the absence of detectable TDP-43 aggregates and are likely to represent upstream pathogenic events in GRN-FTD (Ghoshal et al., 2012; Yang et al., 2025). Our findings support the emerging concept that lysosomal dysfunction is an important molecular consequence of progranulin deficiency and may represent an early and potentially initiating event in pathways relevant to GRN-associated FTD. The observation that reduced CTSD activity exacerbates lysosomal abnormalities in a PGRN-deficient background suggests that impaired lysosomal proteolysis may contribute to molecular and cellular abnormalities that precede overt neurodegeneration. or TDP-43 proteinopathy. These findings further emphasize the importance of investigating early molecular and cellular changes that precede irreversible neuronal damage and may therefore represent more tractable therapeutic targets. Future studies should (i) aim to determine whether restoration of CTSD function or enhancement of lysosomal proteostasis can mitigate disease-relevant phenotypes and delay the emergence of downstream neuropathological abnormalities and (ii) employ models that develop more robust TDP-43 pathology to assess whether impaired CTSD function directly influences TDP-43 aggregation, phosphorylation, mislocalization, or clearance. Establishing such mechanistic links will be necessary to determine whether the molecular phenotypes observed here contribute directly to the neuropathology of FTD.

The present findings also raise the possibility that CTSD may act as an important genetic or functional modifier of GRN-associated disease. While complete CTSD deficiency causes a severe lysosomal storage disorder, our data suggest that even partial alterations in CTSD activity may have meaningful consequences in the context of PGRN deficiency. Future work should investigate whether naturally occurring variation in CTSD expression or activity influences disease onset, progression, or phenotypic heterogeneity in GRN mutation carriers. Such studies may provide additional insight into the mechanisms underlying the marked clinical variability observed among affected individuals.

Overall, our findings demonstrate an important role for PGRN in regulating CTSD activity, lysosomal homeostasis, and protein quality control *in vivo*. These data identify CTSD as a modulator of molecular and disease-relevant phenotypes associated with progranulin deficiency and emphasize the complex interplay between PGRN and CTSD. While these observations are relevant to current models of GRN-associated FTD, they do not establish a direct causal relationship with the neurodegenerative process. Accordingly, the relevance of CTSD-dependent lysosomal dysfunction to the dementing process in GRN-associated FTD should be considered a hypothesis requiring further investigation. Further studies incorporating neuronal survival analyses, region-specific neuropathology, inflammatory profiling, and longitudinal cognitive, detailed lysosomal morphological assessment and social behavioral assessments will be required to determine the extent to which the lysosomal and proteostatic abnormalities described here contribute to neurodegeneration in GRN-associated FTD.

## Materials and methods

### Generation of PRGN and CTSD knockout (KO) mice model

The conditional progranulin knockout (cGrnKO) mouse line was generated in-house as previously described by Beel et al. (2017) and crossed with the PGK-Cre deleter strain (Lallemand et al., 1998) to induce germline recombination of the floxed Grn allele, thereby generating constitutive Grn-deficient mice. These mice were subsequently crossed with heterozygous cathepsin D knockout (CTSD+/−) mice, originally described by Saftig et al. (1995), to generate double-heterozygous (PGRN+/−; CTSD+/−) offspring. Double-heterozygous mice were intercrossed to obtain all possible genotype combinations at the expected Mendelian frequencies. Homozygous Ctsd-deficient (CTSD−/−) offspring were excluded from further analyses because complete cathepsin D deficiency results in early postnatal lethality. Genomic DNA was isolated from ear biopsies using the HotSHOT method (Truett et al., 2000) and used for PCR-based genotyping. The Grn alleles were detected using primers targeting intron 1 (F: 5′-AGAGTAAGTGCCTATCCAAGAACT-3′), exon 8 (F: 5′-CAGAC CCCACTGACCACTGCACTT-3′) and intron 8 (R: 5′-GAACGG AGCACACAGCTAACCCAG-3′). Cycling conditions consisted of an initial denaturation at 95 °C for 5 min, followed by 35 cycles of 95 °C for 30s, 62 °C for 30s, and 72 °C for 30s, with a final extension at 72 °C for 7 min. The expected amplicon sizes were 243 bp for the wild-type allele and 298 bp for the recombined mutant allele. For Ctsd (KO) genotyping, PCR amplification was performed using primers specific for the neomycin-stop cassette inserted into exon 4 of the Ctsd locus (F: 5′-GTTGTCACTGAAGCGGGAAGGGACTGGCTG-3′) and (R: 5′-GCGAACAGTTCGGCTGGCGCGAGCCCCTGA-3′) together with primers targeting an internal control (Cx3) sequence (F: 5′-AAGACTCACGTGGACCTGCT-3′) and (R: 5′-AGGATGTTGA CTTCCGAGTGG-3′). PCR conditions consisted of an initial denaturation at 95 °C for 5 min, followed by 35 cycles of 95 °C for 30s, 58 °C for 20s, and 72 °C for 30s, with a final extension at 72 °C for 7 min. The assay generated a 269 bp amplicon for the Ctsd knockout allele and a 695 bp internal control product, allowing discrimination between wild-type and heterozygous Ctsd knockout animals. Mixed-sex cohorts with an equal male-to-female ratio in each experimental group were used for all experiments.

### Western blot analysis

Brain samples of 18-month-old mice were lysed in RIPA buffer (R0278-500ML, Sigma-Aldrich) supplemented with a protease inhibitor (cOmplete^™^, EDTA-free protease inhibitor cocktail, 1,836,170,001, Sigma-Aldrich), a phosphatase inhibitor (PhosSTOP^™^, 4,906,837,001, Sigma-Aldrich), and 0.1 M phenylmethanesulfonyl fluoride (329–98-6, Sigma-Aldrich). Beads were added to the samples until the tissue was completely covered. The lysed tissues were then homogenized using a tissue homogenizer with the following settings: 3 × 6,500 rpm for 30 s at room temperature, with the homogenized samples kept on ice for 2 min between each cycle. Following homogenization, the lysates were incubated for 30 min at 4 °C, followed by centrifugation at 13,000 rpm for 10 min at 4 °C. The supernatant was collected, transferred to a new tube, and the pellet was discarded. Protein concentration was determined using the Micro BCA^™^ Protein Assay Kit (23,235, Thermo Fisher Scientific) according to the manufacturer’s instructions, and the samples were stored at −80 °C until further use.

Samples were then supplemented with SDS-containing reducing sample buffer (39,000, Thermo Fisher Scientific), denatured for 10 min at 95 °C, and loaded onto Mini-PROTEAN TGX precast gels (Bio-Rad). After electrophoresis (100 V for 1.5 h), the proteins were transferred to a PVDF membrane (Bio-Rad). The membrane was blocked with 5% nonfat dried milk (M7409-1BTL, Sigma-Aldrich) diluted in Tris-buffered saline (T5912-1 L, Sigma-Aldrich) with Tween 20 (P1379-500ML, Sigma-Aldrich) (TBS-T) for 1 h at room temperature to reduce background noise. The membrane was then incubated with primary antibodies overnight at 4 °C (except for housekeeping proteins, where the membrane was incubated with primary antibodies for 1 h at room temperature), followed by 3 washes of 5 min each in TBS-T. After washing, a secondary antibody was applied for 1 h at room temperature. The membrane was then washed 2 times for 5 min each with TBS-T, followed by a final 5-min wash with TBS, and treated with Pierce ECL (32,106, Thermo Fisher Scientific). Images were captured using a chemiluminescence detection system (ImageQuant LAS400). The following antibodies were used for detection: anti-CTSD (ab75852, Abcam), anti-lamp1 (#H4B3, DSHB), anti-lamp2 (ABL-93, Invitrogen), anti-TDP-43 (12892-1-AP, Proteintech), and anti-GAPDH (AM4300, Invitrogen).

### Cell lysate CTSD activity assay

CTSD activity was measured using a CTSD Activity Assay Kit (ab65302, Abcam) according to the manufacturer’s instructions. Briefly, brain samples of 18-month-old mice from different genotypes (WT and PGRN knock-out) were lysed in RIPA lysis buffer (R0278-500ML, Sigma-Aldrich) supplemented with a protease inhibitor (cOmplete^™^, EDTA-free protease inhibitor cocktail, 1836170001, Sigma-Aldrich), a phosphatase inhibitor (PhosSTOP^™^, 4906837001, Sigma-Aldrich), and 0.1 M phenylmethanesulfonylfluoride (329-98-6, Sigma-Aldrich). Following lysis, the samples were centrifuged at 12,000 × g for 10 min at 4 °C. The supernatants were collected, and protein concentrations were determined using the Micro BCA^™^ Protein Assay Kit (23235, Thermo Fisher Scientific) as per the manufacturer’s instructions. An equal amount of protein from each sample was used to measure CTSD activity. The reaction mixture consisted of a CTSD reaction buffer, cell lysates, and an internally quenched fluorescent substrate provided in the kit. To ensure specificity, samples were also measured in the presence of an internal control (included in the kit) to account for any non-specific enzymatic activity. All assays were performed in duplicates using three biological replicates. Data are presented as fold-change relative to WT.

### Measuring mouse PGRN levels

To quantify PGRN levels in brain samples from different genotypes, the Quantikine ELISA Mouse Progranulin Immunoassay kit (MPGRN0, R&D Systems) was used according to the manufacturer’s instructions. Brain tissues from 18-month-old mice were first lysed using RIPA lysis buffer (R0278-500ML, Sigma-Aldrich), which was supplemented with a protease inhibitor (cOmplete^™^, EDTA-free protease inhibitor cocktail, 1836170001, Sigma-Aldrich) and a phosphatase inhibitor (PhosSTOP^™^, 4906837001, Sigma-Aldrich) to prevent protein degradation and dephosphorylation during the extraction process. The lysates were then subjected to centrifugation at 12,000 × g for 10 min at 4 °C to remove cell debris and obtain a clear supernatant containing soluble proteins. Protein concentrations in the supernatants were determined using the Micro BCA^™^ Protein Assay Kit (23,235, Thermo Fisher Scientific) as per the manufacturer’s instructions to ensure equal loading of samples. Following this, equal amounts of protein from each genotype were loaded into the ELISA plate wells. The ELISA procedure involved several incubation steps where specific antibodies in the kit bound to PGRN present in the samples. The antigen–antibody complexes were detected using a secondary antibody conjugated to an enzyme, which catalyzed a colorimetric reaction. The intensity of the color produced, which correlates with the amount of PGRN in the samples, was measured at 450 nm using a Victor Nivo^™^ microplate reader (PerkinElmer). The assay was performed in technical duplicates or triplicates for each sample, with three biological replicates used to ensure data accuracy and reproducibility. The results were then normalized to the WT levels and presented as fold-change relative to the WT controls.

### Immunohistochemistry

#### Immunofluorescence-based immunohistochemistry

To visualize LAMP1 and TDP43 expression in brain samples, tissues from different genotyped 18-month-old mice were fixed in 4% paraformaldehyde in PBS for 30 min and washed 3 times with PBS (5 min each wash). Samples were then blocked for 1 h at room temperature in blocking buffer containing 5% normal donkey serum and 1% Triton X-100 in PBS. After blocking, the samples were incubated overnight at 4 °C in the dark with a primary antibody against LAMP1 and TDP43. The following day, samples were washed 3 times with PBS and subsequently incubated for 60 min at room temperature with the appropriate fluorophore-conjugated secondary antibodies. After 3 PBS washes (5 min each wash), nuclei were counterstained with Hoechst (1/2,000 dilution in PBS) for 15 min at room temperature. The coverslips were then washed 3 times (5 min each wash) with PBS at room temperature, followed by 3 washes with Milli-Q water. Finally, the coverslips were mounted on glass slides, and images were captured using a LEICA SP5 confocal microscope using a 63x objective. Images were analyzed using the widely used non-commercial software ImageJ FIJI (NIH, Bethesda, MD, United States).

#### Chromogenic DAB-based immunohistochemistry

Brains from 18-month-old mice were cut coronally with a cryostate, and sections were collected using the hippocampus as anatomical reference. The sections were thawed to room temperature and rehydrated in 1x TBS-T for 15 min, followed by endogenous peroxidase blocking (Envision Flex Peroxidase-Blocking Reagent, Dako) for 5 min. A serum blocking solution containing normal goat serum (Vectastain Elite ABC HRP Kit, Vector labs, CA, United States) was applied to the slides for 20 min.

Sections were stained overnight at room temperature with the rabbit polyclonal anti-pTDP-43 (409/410, dilution 1:5000, Cosmobio CAC-TIP-PTD-P07, Tokyo, Japan). Sections were then incubated with a biotinylated goat anti-rabbit IgG secondary antibody for 30 min, followed by an avidin–biotin complex (ABC) amplification solution (both Vectastain Elite ABC HRP Kit, Vector labs, CA, USA), also for 30 min. 3,3′-diaminobenzidine (DAB) (Abcam, Cambridge, UK) was used as a chromogen to yield brown reaction products in all sections and counterstaining with hematoxylin, dehydration and mounting was performed in the Leica Autostainer XL and Leica Coverslipper (Leica Microsystems, Heidelberg, Germany). The slides were imaged with a LEICA DM2000 LED microscope, coupled to a Leica DFC 7000 T camera, using the LAS software (Leica Microsystems, Heidelberg, Germany). Whole sections were analyzed under the microscope at 40x magnification and inspected for the presence of pTDP-43-positive neuronal and glial cytoplasmic aggregates. RGB Images were then acquired with a 10x eyepiece and a 20x objective. Regions of interest covered the upper layers of the temporal cortex area (0.28 mm2 micrographs). Images were white balanced to neutralize background. Identical brightness and gain parameters were used for all images.

### Statistics

Unless stated otherwise, experiments were performed using mixed-sex cohorts containing equal numbers of male and female 18-month-old mice. Sex was not included as a factor in the primary statistical analyses and animals were pooled and analyzed together. Sex-stratified analysis was performed for the PGRN ELISA dataset (Supplementary Figure 3). Comparisons between two groups were performed using either a paired ratio t-test or an unpaired Mann–Whitney test, depending on the distribution and pairing of the data. Statistical significance was defined as a *p*-value of less than 0.05, denoted by an asterisk (*). Data are presented as the mean, with error bars representing the standard error of the mean (SEM) calculated from a minimum of three biologically independent experiments. Most data were analyzed using a two-way ANOVA with PGRN genotype (NTG, heterozygous, homozygous) and CTSD genotype (NTG, heterozygous) as between-subject factors and an interaction factor between both genotypes. Moreover, this analysis was followed by a *post hoc* analysis comparing multiple groups using Tukey’s multiple-comparisons tests. However, to provide clarity on the analysis method applied, the specific statistical tests used for each comparison and the composition of the groups are detailed in the corresponding figure legends. All statistical analyses and data visualizations were conducted using GraphPad Prism software (version 7.01, GraphPad Software, Inc., San Diego, CA, United States).

## Data Availability

The raw data supporting the conclusions of this article will be made available by the authors, without undue reservation.
