# Supplementary material for: Progranulin haploinsufficiency disrupts cathepsin D function, resulting in lysosomal abnormalities and elevated TDP-43 levels in the mouse brain
Source: Front Mol Neurosci. 2026 Oct 27;19:1970154. doi: 10.3389/fnmol.2026.1970154 (PMC13748314; doi:10.3389/fnmol.2026.1970154)
Supplement: Supplementary file 1 [file Supplementary_file_1.docx]

**Supplementary Material**

**Supplementary Figure S1: PGRN deficiency results in enhanced levels of LAMP2 proteins but does not change the LAMP2/LAMP1 ratio.** (A) Quantification of LAMP2 protein levels shown in Figure 2A normalized to the housekeeping gene and loading control GAPDH. (B) Quantification of the LAMP2/LAMP1 ratio from the Western blots shown in Figure 2A. Statistical significance was determined using a 2-way ANOVA with Tukey’s Post-hoc multiple group comparison. Data are shown as mean ± SEM of pooled data from six biologically independent mice per group (3 males and 3 females). Statistical significance is indicated as ns (not significant), *P < 0.05, **P < 0.01.

**Supplementary Figure S2: Immunohistochemical analysis of phosphorylated TDP-43 in mouse brain sections.** Representative images of temporal cortex sections from WT, PGRN-/-, and PGRN-/-; CTSD+/- mice immunostained for phosphorylated TDP-43 (pTDP-43, Ser409/410). No phosphorylated TDP-43-positive cytoplasmic inclusions or aggregates were detected in any of the examined genotypes at the analyzed age. These findings indicate that, despite the increase in insoluble TDP-43 observed biochemically (Figure 3), overt pathological TDP-43 aggregation was not evident by immunohistochemistry under the conditions examined. Images were acquired at 400× magnification.

.

**Supplementary Figure S3: PGRN expression is not sex-dependent in our mouse models.** PGRN levels in brain lysates measured by ELISA in different mouse genotypes, including WT (PGRNntg - CTSDntg), heterozygous single knockout (PGRNntg - CTSD+/-), homozygous PGRN single knockout (PGRN-/- - CTSDntg), double knockout of homozygous PGRN and heterozygous CTSD (PGRN-/- - CTSD+/-), heterozygous PGRN single knockout (PGRN+/- - CTSDntg) and double knockout heterozygous PGRN and heterozygous CTSD (PGRN+/- - CTSD+/-) of (A) male mice, (B) female mice. Statistical significance was determined using a 2-way ANOVA with Tukey’s Post-hoc multiple groups comparison. Data are shown as mean ± SEM of three biologically independent mice per group. Statistical significance is indicated as ns (not significant), *P < 0.05.
